# DCAF26, an Adaptor Protein of Cul4-Based E3, Is Essential for DNA Methylation in *Neurospora crassa*

**DOI:** 10.1371/journal.pgen.1001132

**Published:** 2010-09-23

**Authors:** Hui Xu, Jiyong Wang, Qiwen Hu, Yun Quan, Huijie Chen, Yingqiong Cao, Chunbo Li, Ying Wang, Qun He

**Affiliations:** 1 State Key Laboratory of Agrobiotechnology, College of Biological Sciences, China Agricultural University, Beijing, China; 2 Biomedical Analysis Center, Tsinghua University, Beijing, China; Medical Research Council Human Genetics Unit, United Kingdom

## Abstract

DNA methylation is involved in gene silencing and genome stability in organisms from fungi to mammals. Genetic studies in *Neurospora crassa* previously showed that the CUL4-DDB1 E3 ubiquitin ligase regulates DNA methylation via histone H3K9 trimethylation. However, the substrate-specific adaptors of this ligase that are involved in the process were not known. Here, we show that, among the 16 DDB1- and Cul4-associated factors (DCAFs) encoded in the *N. crassa* genome, three interacted strongly with CUL4-DDB1 complexes. DNA methylation analyses of *dcaf* knockout mutants revealed that *dcaf26* was required for all of the DNA methylation that we observed. In addition, histone H3K9 trimethylation was also eliminated in *dcaf26^KO^* mutants. Based on the finding that DCAF26 associates with DDB1 and the histone methyltransferase DIM-5, we propose that DCAF26 protein is the major adaptor subunit of the Cul4-DDB1-DCAF26 complex, which recruits DIM-5 to DNA regions to initiate H3K9 trimethylation and DNA methylation in *N. crassa*.

## Author Summary

DNA associates with histones to form chromatin in eukaryotes. Epigenetics refers to DNA and histone modifications in chromatin that persist from one cell generation to the next, controlling gene expression and genome stability. These epigenetic changes are crucial for the development and differentiation of the various cell types in eukaryotes. In this study, we identified DCAF26 as a crucial regulator of DNA methylation. Inactivation of this gene in *N. crassa* resulted in loss of both DNA methylation and histone H3K9 trimethylation. We found that the resulting severe defects in the development and growth of *dcaf26* mutants were similar to those found in *cul4*, *ddb1*, and *dim-5* mutants, suggesting that these four genes function in the same pathway. Furthermore, we showed that DCAF26 functioned as an adaptor protein for the Cullin4-DDB1 complex to recruit the histone methyltransferase DIM-5 and regulate trimethylation of histone H3K9, which marks DNA for methylation. Our results reveal important roles for DCAF26 in H3K9 trimethylation and DNA methylation in *N. crassa* and suggest a conserved mechanism for DNA methylation in eukaryotic organisms.

## Introduction

The Cul4-DDB1 complex, a major class of cullin-RING ubiquitin ligases (CRLs), is evolutionarily conserved from yeasts to humans. Previous studies have indicated that Cul4-DDB1-regulated ubiquitination is linked to multiple processes, such as cell cycle regulation, DNA replication licensing, DNA repair, and gene expression processes [1]–[3]. In the CRLs, cullin associates with substrates via adaptor molecules in the N terminus, and interacts with the E2 enzyme via the RING finger protein Hrt1/ROC1/Rbx1 in the C terminus [4]. Substrate-specific adaptors, such as the F-box-containing proteins in SCF complexes and the BTB domain-containing proteins in Cul3-based ubiquitin ligases, determine substrate-specific ubiquitination in many biological processes [5]. Although the different structural states of DDB1 may allow it to directly recruit substrates to the Cul4-based E3 platform, studies have demonstrated that ubiquitination of several characterized CUL4-DDB1 substrates requires additional substrate-specific adaptors [6], [7].

Recent studies showed that a class of adaptors called DCAFs (DDB1- and Cul4-associated factors) [4] are employed by Cul4-based E3 ligases to identify specific proteins for ubiquitination. Most DCAFs are WD40-containing proteins with relatively conserved “WDXR” motifs that interact with DDB1 protein. However, several DCAF proteins lacking this conserved motif are still able to bind DDB1 in vivo [3], [4], [8], [9].

Among the well-characterized DCAF proteins, mammalian WDR5 and RBBP5 are essential components of the histone methyltransferase complex that methylates histone H3 on lysine 4 (H3K4) [10]–[12]. Further studies showed that Cul4-DDB1 can interact with WDR5 and RBBP5 and regulate histone H3K4 methylation. Down regulation of each of these genes by siRNA severely reduces the tri- and monomethylation of histone H3K4, but not H3K4 dimethylation [13]. Interestingly, inactivation of Cul4 or DDB1 also causes a significant inhibition of histone H3K9 and H3K27 trimethylation [13]. However, none of these DCAFs were shown to be involved directly in DNA methylation in eukaryotes.

In fission yeast, the Cul4-Rik1 E3 ubiquitin ligase associates with the histone methyltransferase Clr4 on heterochromatic regions to methylate histone H3K9, contributing to heterochromatin assembly and maintenance [14]. The catalytic activity of Cul4 is required for its proper function in heterochromatin formation. This study suggests that the activity of Cul4-based E3 ligase is required for histone H3K9 methylation. In addition, a WD-40-containing protein, Raf1/Dos1/Clr8/Cmc1, is required for histone H3K9 methylation and heterochromatin formation in *S. pombe* [15]–[18]. Thus, these studies imply that Raf1/Dos1/Clr8/Cmc1 functions as an adaptor protein associated with Cul4-Rik1 complex in *S. pombe*.

We previously demonstrated that Cul4-DDB1 E3 ligase is essential for DNA methylation in *N. crassa* by regulating histone H3K9 trimethylation [19]. These results suggest that Cul4-DDB1 ubiquitin ligase is required for epigenetic control in higher eukaryotes. However, the substrate-specific adaptors of Cul4-DDB1 E3 ligase and the requirement of DCAFs as the substrate adaptors in DNA methylation are unknown in *N. crassa*.

Here, we identified three DCAFs that could strongly interact with DDB1 protein in vivo, out of 16 candidates in the *N. crassa* genome. DNA methylation analysis in knockout strains of the 12 *dcaf* genes showed that *dcaf26* was essential for DNA methylation in *N. crassa*. The *dcaf26* deletion mutant also lost histone H3K9 trimethylation. Our protein interaction results suggest that DCAF26 functions as an adaptor subunit of the Cul4-DDB1-DCAF26 complex by recruiting histone methyltransferase DIM-5 to DNA methylation regions in *N. crassa*. In addition, we show that the interaction of DCAF26 with DDB1 was required to enable the Cul4-DDB1-DCAF26-DIM-5 complex to regulate H3K9 trimethylation and DNA methylation in this organism.

## Results

### Comparative analysis of *N. crassa* DCAF proteins

**Figure 1:**
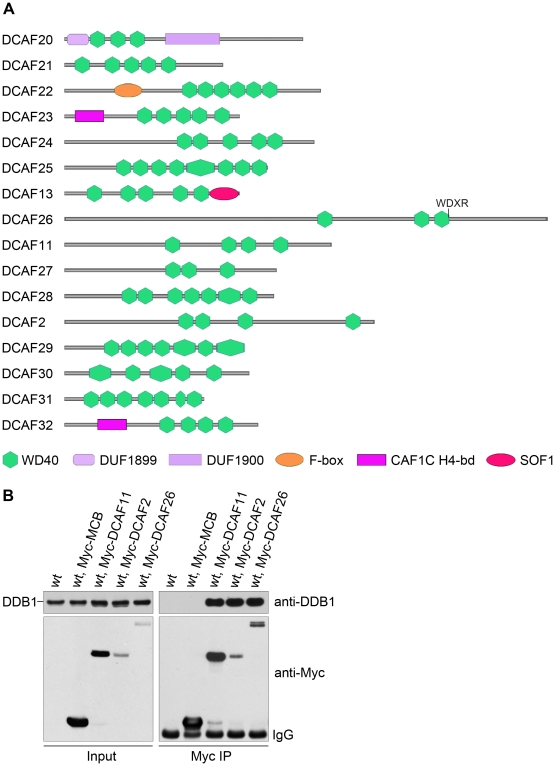
Putative DDB1- and Cul4-associated factors (DCAFs) in *N. crassa*. (A) Schematic representation of 16 putative DCAFs with predicted domains. (B) Interactions between Myc-tagged DCAFs and DDB1 protein. c-Myc antibody was used for immunoprecipitation, followed by western blot analysis using DDB1 or c-Myc antibodies. The wild-type strain and wild-type expressing Myc-tagged MCB (the regulatory subunit of cAMP-dependent protein kinase) strain were used as negative controls.

We searched the *N. crassa* genome for WD-40-containing proteins with WDXR and DxR motifs (also known as a DWD motif). The conserved 16 amino acid sequence [IFVL]-[IFVL]-[AGST]-[AGST]-[AGST]-x-[DE]-x(2)-[IFVL]-x-[IFVL]- [WY]-[DE]-[IFVL]-[RK] [20] was used as the seed to search for WD-40-containing proteins. Five putative DCAFs met the criteria (Table 1). Based on the sequences of hundreds of DCAF proteins from yeast to human that have been identified by protein interaction experiments and bioinformatics analysis, we identified another 11 putative DCAF proteins (Table 1). In total, we identified 16 putative DCAF proteins in the *N. crassa* genome (Figure 1A).

**Table 1:** Putative DCAFs in *Neurospora crassa*.

	DCAFs	NCU Number	Gene name in *Neurospora* database	Homolog	WDXR motif	Required for DIM-2-dependent DNA methylation
DWD motif	DCAF20	00202.3	coronin-6	Coronin	1	×
	DCAF21	01389.3	mitogen-activated protein kinase organizer 1	MAPKO1	1	×
	DCAF22	06483.3	F-box and WD repeat-containing protein	FBW7	0	×
	DCAF23	06679.3	histone acetyltransferase type B subunit 2	CAF1C	1	×
	DCAF24	07724.3	mitochondrial division protein 1	MDV1	2	×
homologue search	DCAF25	00794.3	ribosome biogenesis protein Rsa4	NLE1/Notchless	0	×
	DCAF13	01595.3	SOF1	DCAF13/WDSOF1	0	Not tested
	DCAF26	01656.3	conserved hypothetical protein	Raf1/Clr8/Dos1/Cmc1	1	√
	DCAF11	02151.3	WD repeat containing protein 23	DCAF11/WDR23	2	×
	DCAF27	02729.3	transducin family protein	PWP1	0	Not tested
	DCAF28	03244.3	WD repeat protein	WDR5	1	×
	DCAF2	03668.3	WD domain-containing protein	DCAF2/CDT2	2	×
	DCAF29	04534.3	nuclear migration protein nudF	Nudf/LIS1	1	×
	DCAF30	05426.3	WD repeat protein	WDR39/CIAO1	0	Not tested
	DCAF31	05797.3	U5 snRNP complex subunit	WDR57	3	×
	DCAF32	09521.3	ribosome biogenesis protein	GRWD1/RRB1	2	Not tested

To identify the true DCAFs in *N. crassa*, we examined the interactions between DDB1 and the predicted DCAF proteins. We first made constructs in which the DCAF ORF was under the control of a quinic acid (QA)–inducible promoter [21]. To facilitate the detection of DCAF expression, five copies of the c-Myc epitope and six histidine residues [22] were inserted into the N terminus of the DCAF ORF. The constructs were then transformed into a wild-type strain and Myc-DCAF expression in the resulting transformants in the presence of QA was confirmed by western blot analysis using the c-Myc antibody. Afterwards, immunoprecipitation assays were used to examine the interactions between Myc-DCAF proteins and DDB1 in the transformants. As shown in Figure 1B, three Myc-tagged DCAFs, DCAF11, DCAF2, and DCAF26, interacted strongly with the DDB1 protein in vivo. Our DDB1 antibody depleted with tissue of the *ddb1^KO^* strain specifically recognized a 128-kDa band in the wild-type strain, but not in the *ddb1* mutant (Figure S1). DCAF11 and DCAF2 had two conserved WDXR motifs, while DCAF26 had only one conserved WDXR motif (WNVR). In contrast, only weak interactions were detected between DDB1 and the other Myc-DCAFs (Figure S2). These results suggest that DCAF11, DCAF2, and/or DCAF26 could be the adaptor(s) in the Cul4-DDB1 E3 ligase complex in *N. crassa*.

### Knockout of *N. crassa dcaf* genes

Our previous study showed that the Cul4-DDB1 E3 complex is required for DNA methylation in *N. crassa* [19]. To investigate the function of the putative DCAFs in DNA methylation, we tried to generate deletion strains of the 16 candidate *dcaf* genes by gene replacement in the *ku70^RIP^* strain. However, we could not obtain the homokaryotic deletion strains of four genes (NCU01595, NCU02729, NCU05426, and NCU09521), suggesting that they are important for cell viability in *N. crassa*. Several independent homokaryotic knockout strains of the other 12 *dcaf* genes were obtained by microconidia purification. PCR analysis confirmed the integration of the knockout cassette at the endogenous *dcaf* locus, and no *dcaf* ORF signals were detected in these knockout mutants.

### DCAF26 is essential for DNA methylation

**Figure 2:**
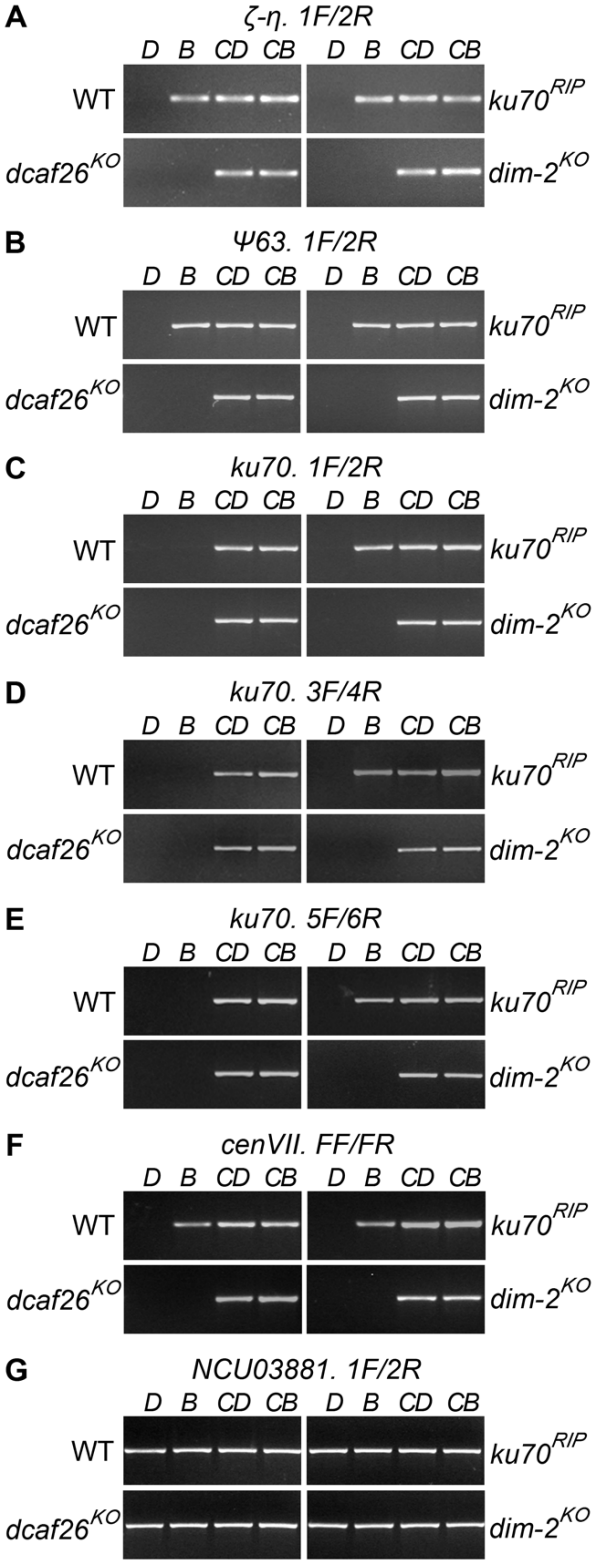
DCAF26 is essential for *N. crassa* DNA methylation. DNA methylation in the wild-type strain (WT) and *ku70^RIP^*, *dcaf26^KO^*, and *dim-2^KO^* strains on the (A) ζ-η region, (B) ψ63 region, (C–E) the *ku70* and *ku70^RIP^* loci, and (F) the centromeric VII region detected by methylation-sensitive restriction digestion. (G) Digested genomic DNA served as a template for PCR of the NCU03381 region, which was covered by primer pairs and had no DpnII site. The knockout mutants were in a *bd ku70^RIP^* background. Genomic DNA digested by 5mC-sensitive BfuCI (*B*) or its 5mC-insensitive isoschizomer, DpnII (*D*), was amplified by PCR with the labeled primers. Untreated genomic DNA as template for PCR was used as the control (*CD* or *CB*).

To identify the DCAF protein(s) required for DNA methylation, we measured the cytosine methylation states of all homokaryotic *dcaf* mutants. Genomic DNA of the wild-type strain and *ku70^RIP^*, *dim-2^KO^*, and *dcaf* mutants was digested with DpnII or BfuCI (Sau3AI). Undigested and digested DNA was then used as templates for PCR. Three representative methylated regions, the *ku70^RIP^* locus, ζ-η, and ψ63, were examined in the wild-type strain, *ku70^RIP^*, *dim-2^KO^*, and *dcaf* mutants. As shown in Figure 2, no PCR products were detected with DpnII- or BfuCI-digested genomic DNA as templates in the *dcaf26^KO^* mutant, the same as for the *dim-2^KO^* mutant. To further confirm these results, we tested the known methylation region on centromere VII of *N. crassa*. As expected, methylation on this DNA region was also lost in the *dcaf26^KO^* mutant (Figure 2), indicating that DCAF26 plays an essential role in DNA methylation. In contrast, the other 11 homokaryotic *dcaf* mutants exhibited normal cytosine methylation patterns at ζ-η and ψ63 regions, the same as those in the wild-type strain (Figure S3). This demonstrated that they were not essential for DIM-2–dependent DNA methylation (Table 1). Taken together, these results suggest that DCAF26 was a key regulator of DNA methylation in *N. crassa*.

### DCAF26 is required for H3K9 trimethylation

**Figure 3:**
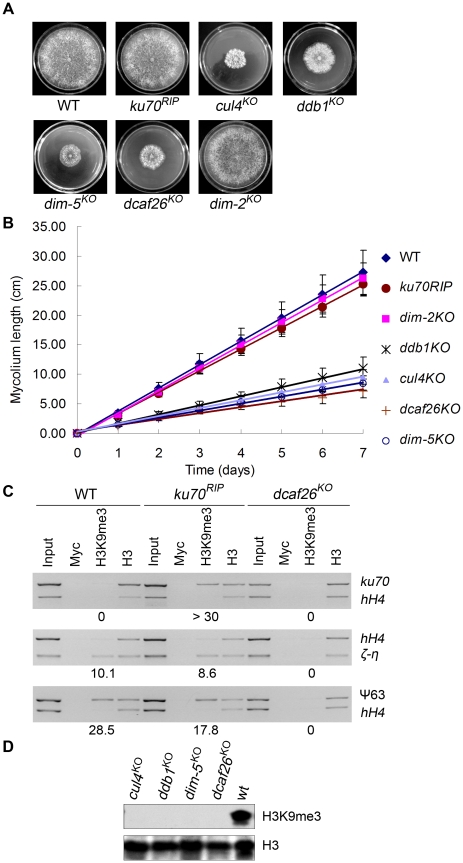
DCAF26 is essential for histone H3K9 trimethylation. (A) Dense, cauliflower-like growth patterns of *dcaf26^KO^*, *cul4^KO^*, *ddb1^KO^*, and *dim-5 ^KO^* strains on plates with minimal media (30°C, 32h). (B) Growth rate of the wild-type, *ku70^RIP^*, *dcaf26^KO^*, *cul4^KO^*, *ddb1^KO^*, *dim-5^KO^*, and *dim-2^KO^* strains, measured at 25°C using the racetube assay in constant darkness. (C) Loss of histone H3K9 trimethylation at *ku70^RIP^*, ζ-η, and ψ63 regions in the *dcaf26^KO^* strain. Levels of H3K9 trimethylation at the indicated locations were determined by ChIP assay. Myc antibody was used as the negative control, and H3 antibody was used as the positive control and as the control for integrity of the nucleosome structure. (D) Western blot analysis of global H3 and H3K9 trimethylation in the wild-type strain and mutants.

In *N. crassa*, proper DNA methylation depends on histone H3K9 trimethylation, which is controlled by the histone methyltransferase DIM-5 [23], [24]. When we compared the growth and developmental phenotypes of the *dcaf* knockout strains to those of the wild-type strain and *cul4^KO^*, *ddb1^KO^*, and *dim-5^KO^* mutants, we found that the *dcaf26* mutant exhibited dense, cauliflower-like growth patterns with abnormal hyphae and asexual spores on plates (Figure 3A). This was similar to the phenotypes of the *cul4*, *ddb1*, and *dim-5* deletion strains [19] (Figure 3A). The *dcaf26^KO^* mutant also exhibited slow growth rates on racetubes compared to that of the wild-type strain (Figure 3B); these rates were similar to those of the *cul4^KO^*, *ddb1^KO^*, and *dim-5^KO^* strains. These results suggest that DCAF26 affected DNA methylation by regulating H3K9 trimethylation in the same pathway as the Cul4-DDB1 complex. Therefore, we next examined H3K9 trimethylation levels by chromatin immunoprecipitation (ChIP) in the wild-type strain, the *ku70^RIP^* strain, and the *dcaf26* mutant. Chromatin samples were immunoprecipitated with antibody against trimethylated Lys9 of histone H3 and analyzed by PCR with primers targeted to methylated DNA regions. As shown in Figure 3C, trimethylated H3K9 was associated with the methylated *ku70^RIP^* region in the *ku70^RIP^* strain, whereas trimethylated H3K9 at the *ku70^RIP^* region was abolished in the *dcaf26* mutant. Similarly, trimethylated H3K9 at the ζ-η and ψ63 regions in the *ku70^RIP^* strain was observed at levels comparable to the wild-type strain (Figure 3C). In contrast, H3K9 trimethylation was lost in the *dcaf26* mutant (Figure 3C). Taken together, these data indicate that *N. crassa* DCAF26 was required for trimethylation of histone H3K9 at methylated DNA regions.

To investigate the effect of DCAF26 protein on global trimethylated H3K9 in *N. crassa*, we tested the levels of histone H3 or trimethylated H3K9 using western blot analysis in wild-type, *dcaf26^KO^*, *cul4^KO^*, *ddb1^KO^*, and *dim-5^KO^* strains. As shown in Figure 3D, all of the strains had similar levels of histone H3. In contrast to the robust H3K9 trimethylation in the wild-type strain, very little trimethylated H3K9 was detected in *dcaf26^KO^*, *cul4^KO^*, *ddb1^KO^*, *dim-5^KO^* strains. Together, these results strongly suggest that DCAF26 protein was required for histone H3K9 trimethylation.

### DCAF26 is the key component for recruiting DIM-5 to the Cul4-DDB1 complex

**Figure 4:**
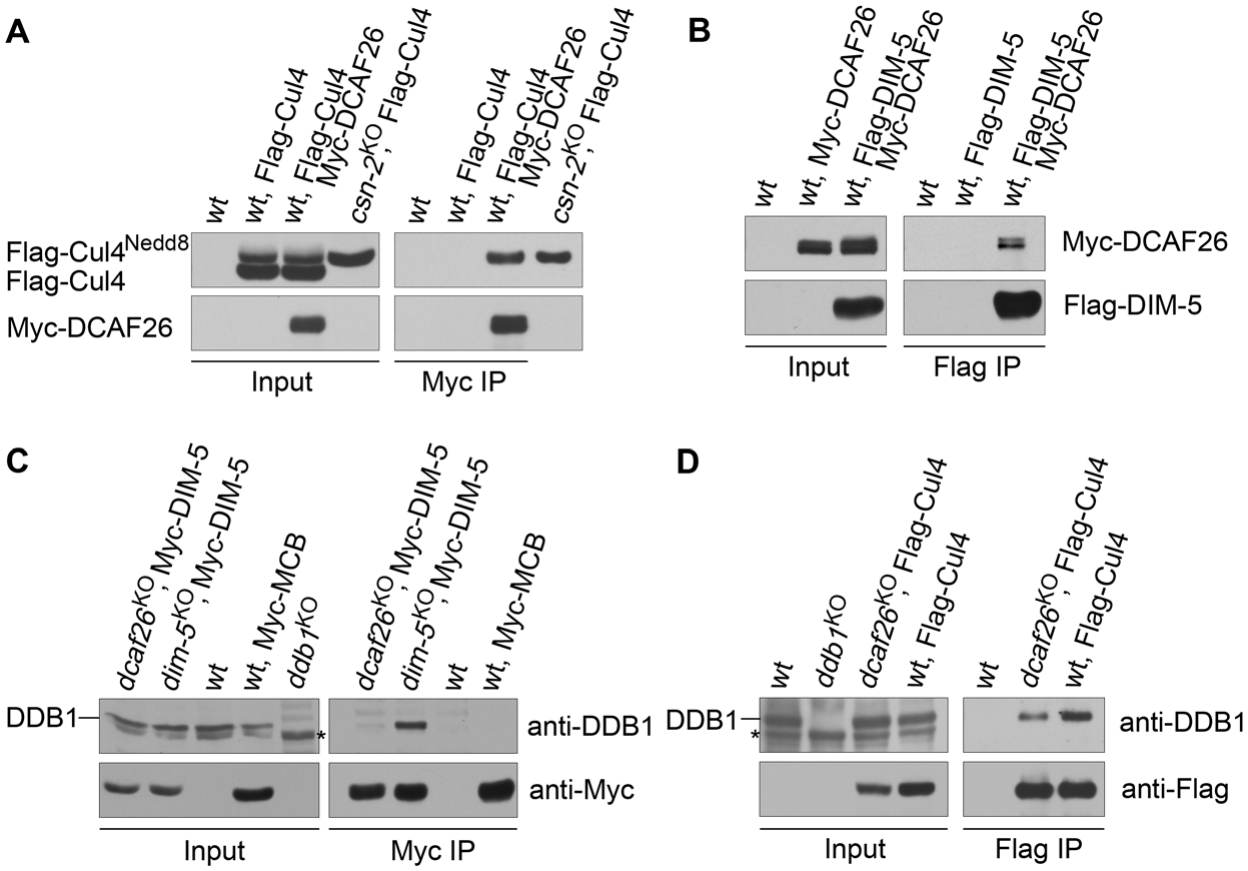
DCAF26 is the key component for recruiting DIM-5 to the Cul4-DDB1 complex. (A) Immunoprecipitation assay using Myc antibody showing the association between Myc-DCAF26 and Flag-Cul4. Myc-DCAF26 interacted with neddylated rather than unneddylated Flag-Cul4. The wild-type strain and the wild-type strain expressing Flag-Cul4 only were used as negative controls. Constitutively hyperneddylated Flag-Cul4 expressed in the *csn-2^KO^* strain was used as the positive control for neddylated Flag-Cul4 (lanes 4 and lane 8). (B) The interaction between Myc-DCAF26 and Flag-DIM-5. (C) Immunoprecipitation assay with Myc antibody showing that DDB1 formed a complex with Myc-DIM-5 in the *dim-5^KO^* complemented strain with expressing Myc-DIM-5, but not in the *dcaf26^KO^* strain. (D) Interactions of Flag-Cul4 and DDB1 independent of DCAF26 protein. Asterisks indicate nonspecific bands detected by anti-DDB1 serum.

Having identified DCAF26 as a DCAF protein that interacted strongly with DDB1 protein, we next tested whether DCAF26 was a key component in the Cul4-DDB1-DIM-5 complex for DNA and histone H3K9 methylation. We did so by performing an immunoprecipitation assay to detect interactions between Myc-DCAF26 and Flag-Cul4 and between Myc-DCAF26 and Flag-DIM-5. As shown in Figure 4A, Myc-tagged DCAF26 specifically interacted with one of two Flag-Cul4 species (neddylated/unneddylated Cul4). To determine whether DCAF26 preferentially interacts with neddylated or unneddylated Cul4, we loaded protein extract from a *csn-2^KO^* strain, in which Cul4 remained in a hyperneddylated state, side by side with Myc-DCAF26/Flag-Cul4 to show that the upper band in the input and the Myc-DCAF26-interacting Cul4 band were neddylated Cul4 (Figure 4A), similar to the association between DIM-5 and neddylated Cul4 species in *N. crassa* [19]. We then examined the interactions between DCAF26 protein and DIM-5. As expected, the Flag antibody pulled down the Myc-tagged DCAF26 in the strain that coexpresses Flag-DIM-5 and Myc-DCAF26 (Figure 4B), indicating that DCAF26 was a component of the Cul4-DDB1-DIM-5 complex. These data explain the similar phenotypes in *dcaf26^KO^*, *cul4^KO^*, *ddb1^KO^*, and *dim-5^KO^* mutants.

We next investigated the function of DCAF26 in this complex. We checked the interactions of DDB1-DIM-5 in the *dcaf26^KO^* strain and in a *dim-5^KO^*, qa-Myc-DIM-5 transformant. As shown in Figure 4C, the interactions between DDB1 and DIM-5 were severely impaired in the *dcaf26^KO^* strain, while DIM-5 strongly interacted with DDB1 in the presence of DCAF26. This indicates that DCAF26 was required for recruiting DIM-5 to the Cul4-DDB1 complex. Furthermore, the interaction between DDB1 and Cul4 was not affected in the *dcaf26^KO^* mutant (Figure 4D), confirming that DCAF26 was an adaptor protein in the Cul4-DDB1-DIM-5 complex.

### Interactions between DCAF26 and DDB1 are essential for DNA and H3K9 methylation

**Figure 5:**
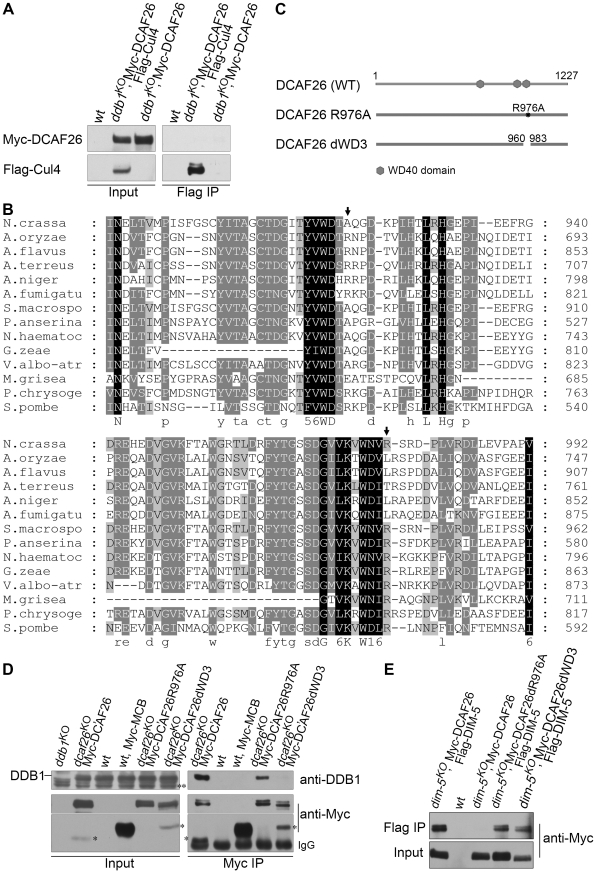
WD3 region of DCAF26 is necessary for binding between DCAF26 and DDB1. (A) Immunoprecipitation assay with Flag antibody showing that Flag-Cul4 failed to form a complex with Myc-DCAF26 in the *ddb1^KO^* strain. (B) Amino acid alignment of the conserved WD40 regions and WDXR motifs of different fungi DCAF26 homologs. The identical residues among different homologs are shaded. Arrows point out the arginine in the WDXR motif. (C) Graphic diagrams showing the domain structure of DCAF26 and different DCAF26 internal point mutations or deletion mutants. (D) Immunoprecipitation assay with c-Myc antibody showing that Myc-DCAF26 failed to interact with DDB1 in the DCAF26dWD3 mutant, but not in the DCAF26R976A mutant, compared to the strong interaction between DDB1 and wild-type DCAF26. Double asterisks indicate nonspecific bands detected by anti-DDB1 serum. Asterisks indicate degraded species of Myc-DCAF26. (E) Immunoprecipitation assay with Flag antibody showing that the mutant Myc-DCAF26 in the DCAF26R976A and DCAF26dWD3 strains still interacted with Flag-DIM-5.

The finding that *N. crassa* DCAF26 interacts with DDB1 and neddylated Cul4 to form a complex prompted us to investigate the functional importance of the interaction between DCAF26 and DDB1. As shown in Figure 5A, DCAF26 and Cul4 protein interactions were totally abolished in the *ddb1^KO^* mutant, suggesting that DDB1 served as a bridge between Cul4 and DCAF26 to form the complex. This result suggests that DDB1, DCAF26, and their interactions contributed the H3K9 and DNA methylation functions of the Cul4-DDB1-DCAF26-DIM-5 complex.

DCAF26 (NCU01656.3) contains some interesting sequence features, including one WDXR motif (WNVR) and WD-40 repeat regions. When the DCAF26 protein sequence was queried in a blast search against protein databases, DCAF26 was found to be similar to various fungal homologs. Many DCAF26 homologs contain one WDXR motif and one WDTA, or another WDXR motif located separately between consecutive “propeller blade” folds of the protein (Figure 5B). *N. crassa* DCAF26 protein contains one WDTA (aa 917–920) motif and one conserved WDXR (WNVR973–976) motif (Figure 5B).

To determine which domains of DCAF26 are involved in the DCAF26-DDB1 interaction, we mutated arginine 976 to alanine in the WNVR motif (Figure 5C) in qa-Myc-His-DCAF26 construct. As shown in Figure 5D, this mutation of DCAF26 reduced binding with DDB1 compared to the binding between wild-type DCAF26 and DDB1 in *dcaf26^KO^* transformants. However, this DCAF26 mutation did not affect the interactions between DCAF26R(976)A and DIM-5 (Figure 5E). We then deleted 24 amino acids (960–983) from DCAF26; this amino acid stretch included the WNVR motif and the third WD-40 domain (Figure 5C). As shown in Figure 5D, the interaction of DCAF26dWD3 with DDB1 was totally abolished in *dcaf26^KO^* qa-Myc-DCAF26dWD3 transformants. However, this DCAF26 deletion did not affect interactions between DCAF26dWD3 and DIM-5 (Figure 5E). These results indicate that the WNVR motif and adjacent amino acids were important for interactions with DDB1, not interactions with DIM-5.

**Figure 6:**
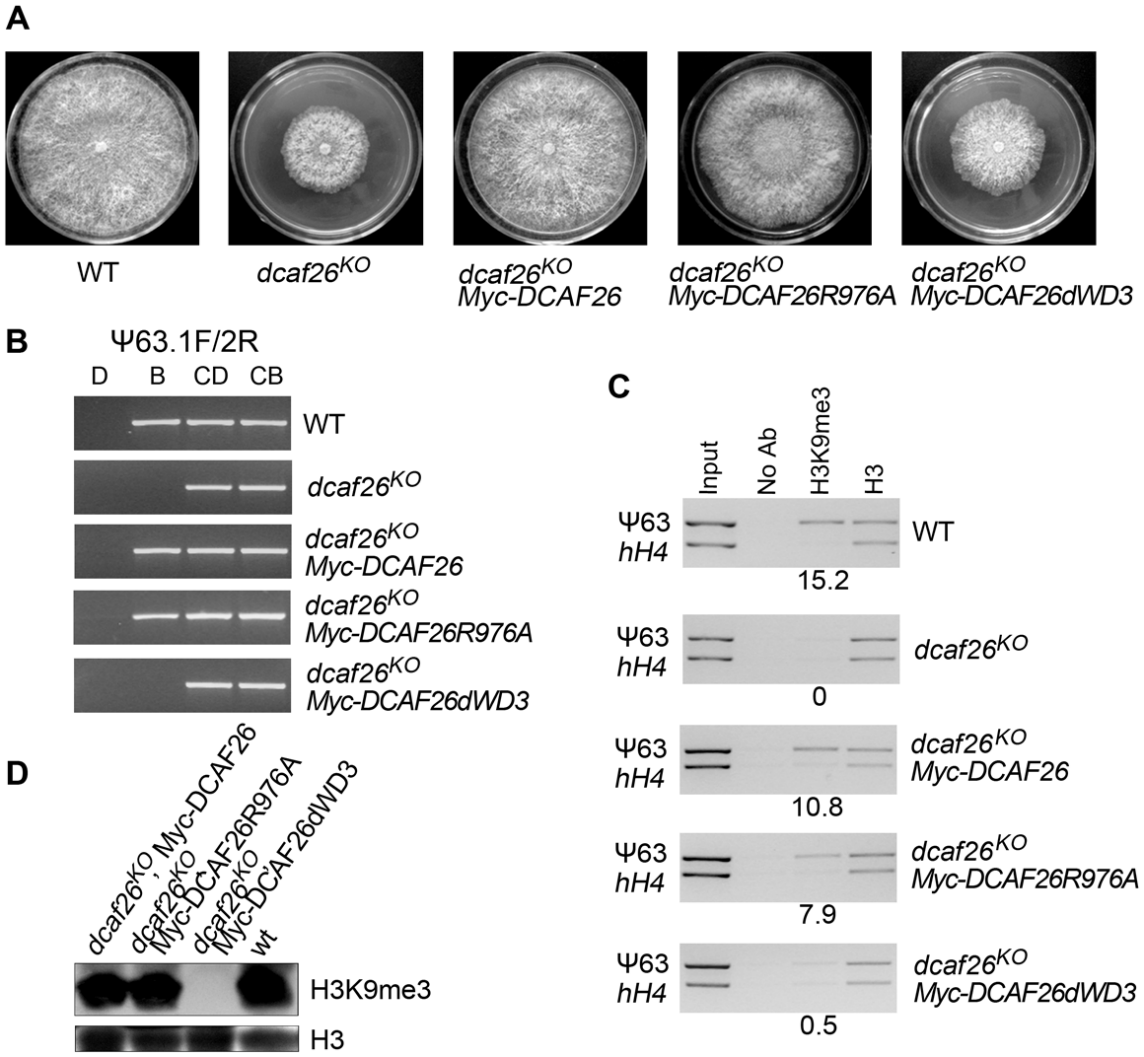
Interactions between DCAF26 and DDB1 are required for proper functioning of the Cul4-DDB1-DCAF26-DIM-5 complex. (A) Growth and developmental defects of the *dcaf26^KO^* strain were complemented by wild-type Myc-DCAF26 or mutated Myc-DCAF26R976A, but not Myc-DCAF26dWD3. (B) Loss of DNA methylation in *dcaf26^KO^* was rescued by expressing Myc-DCAF26 or Myc-DCAF26R976A, but not Myc-DCAF26dWD3. (C) Loss of H3K9 trimethylation at the ψ63 region in the *dcaf26^KO^* strain was rescued by expressing Myc-DCAF26 or Myc-DCAF26R976A, but not Myc-DCAF26dWD3. (D) Western blot analysis showing that the loss of global H3K9 trimethylation in *dcaf26^KO^* was restored by Myc-DCAF26 or Myc-DCAF26R976A, but not Myc-DCAF26dWD3. H3 antibody was used as the positive control and as the control for integrity of the nucleosome structure.

To further examine the function of the interaction between DCAF26 and DDB1, we investigated the function of these mutated DCAF26 in *dcaf26^KO^* transformants. As shown in Figure 6A, the expression of Myc-tagged wild-type DCAF26 fully rescued the growth and developmental defects of the *dcaf26^KO^* mutant, resulting in a similar growth rate as the wild-type strain on plates. Importantly, the DNA methylation (Figure 6B) and the H3K9 trimethylation (Figure 6C and 6D) in the *dcaf26^KO^*, qa-Myc-His-DCAF26 transformant were also restored. The expression of Myc-DCAF26R(976)A mutant protein can restore the growth and developmental phenotypes (Figure 6A), as well as DNA methylation (Figure 6B) and H3K9 trimethylation (Figure 6C and 6D) of *dcaf26^KO^* mutant. These results indicated that the weak interaction of DCAF26-DDB1 is sufficient for the formation of Cul4-DDB1-DCAF26-DIM-5 complex. In contrast, the expression of Myc-DCAF26dWD3 mutant protein failed to rescue the growth and developmental phenotypes (Figure 6A) and the defects of DNA methylation (Figure 6B) and H3K9 trimethylation (Figure 6C and 6D) of *dcaf26^KO^* mutant, indicating that the interaction of DCAF26-DDB1 was required for the proper function of Cul4-DDB1-DCAF26-DIM-5 complex. Taken together, these results further demonstrate that DCAF26 is a critical component in the Cul4-DDB1 ubiquitin ligase in *N. crassa*.

### Purification of DCAF26 and identification of DCAF26-associated proteins

Recent studies demonstrate the biochemical function of the DCAF protein DDB2 as a substrate receptor for XPC ubiquitination in DDB1-DDB2-CUL4A-Rbx1 complex in human cell lines [25]. Since we have demonstrated that DCAF26 is required for recruiting DIM-5 to the Cul4-DDB1 complex, we wondered whether there are other substrates associated with the complex.

**Figure 7:**
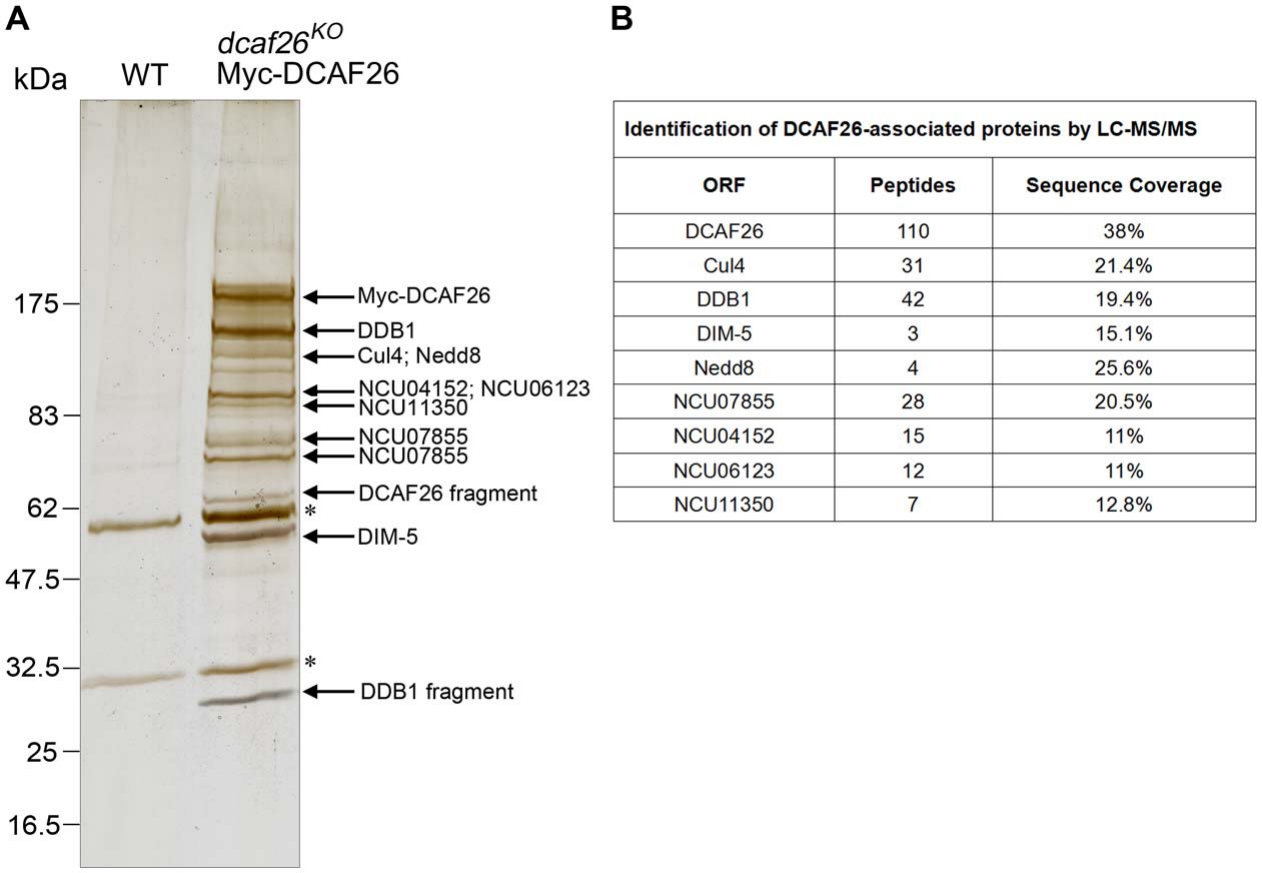
Identification of DCAF26-associated proteins. (A) Silver-stained SDS-PAGE showing the two-step purification of *N. crassa* DCAF26. Wild-type strain was used as the negative control. Asterisks indicate the two IgG bands. (B) Eight DCAF26-associated proteins were identified by LC-MS/MS.

To better understand the substrate adaptor role of DCAF26 for the Cul4-DDB1 E3 ligase, we attempted to purify this protein in *N. crassa*. To do so, we expressed Myc-His-DCAF26 protein by inoculating the *dcaf26^KO^*, qa-Myc-His-DCAF26 strain in liquid media containing quinic acid. Then Myc-His-DCAF26 protein was purified by nickel-column followed by immunoprecipitation using the c-Myc monoclonal antibody. As shown in Figure 7A, several major protein bands were specifically observed in the Myc-His-DCAF26 sample but not in the negative control (WT lane). The LC-MS/MS analysis of excised gel bands led to the identification of five well-known proteins, DCAF26, Cul4, DDB1, Nedd8, DIM-5 (the histone H3K9 methyltransferase in *N. crassa*) (Figure 7A and 7B). All proteins were represented with three or more peptides. The coexistence of Nedd8 peptides with Cul4 was consistent with previous results showing that DCAF26 preferentially interacted with neddylated Cul4 proteins in vivo, further confirming that DCAF26 is a key component of Cul4-DDB1 E3 ligase. In addition, we also identified other four DCAF26 co-purified proteins (Figure 7A and 7B) encoded by NCU07855, NCU04152, NCU06123, and NCU11350, respectively. Among these proteins, NCU04152 (DIM-7) is also required for DNA methylation and H3K9 trimethylation (Figure S4). Since the submission of this paper, similar results were recently shown by Lewis et al. [26]. Furthermore, the absence of ubiquitin peptides in the purification products suggests that either the ubiquitinated substrate is released immediately from Cul4-DDB1 complex or its ubiquitination level is too low to be detected.

## Discussion

Cul4-based E3 ubiquitin ligases are evolutionarily conserved multifunctional complexes in eukaryotes. In this study, using a genetic screen to find genes that encode proteins interacting with DDB1 and Cul4 in *N. crassa*, we identified DCAF26 as a new component required for DNA methylation. *dcaf26^KO^* mutants exhibited the loss of all known DNA methylation and H3K9 trimethylation. Our data demonstrate that DCAF26 interacts with DDB1, which in turn recruits DIM-5 via the Cul4-DDB1-DCAF26 complex to regulate H3K9 trimethylation and DNA methylation.

### DCAF26 is an essential component in the Cul4-DDB1 complex for recruiting DIM-5 to regulate H3K9 trimethylation

Recent studies demonstrated that three proteins, DIM-5 [23], HP1 [27], and DIM-2 [28], are essential for DNA methylation in *N. crassa*, which function in histone H3K9 trimethylation and DNA methylation. To understand the regulation of DNA methylation in *N. crassa*, we sought to identify the proteins operating upstream of DIM-5. Previously, we showed that DDB1 and Cul4 function in an early step of DNA methylation by forming a complex with DIM-5 to regulate H3K9 trimethylation in *N. crassa* [19]. To determine the precise function of this complex, we sought to identify the DDB1- and Cul4-associated factors (DCAFs) involved in this pathway. In mammalian cells, the DCAF protein WDR5 is a core component of the histone methylation complex essential for H3K4 methylation [12], and loss of WDR5 specifically affects tri- and monomethylated H3K4 [13]. However, there is limited evidence to suggest that histone H3 methylation is directed by DCAF protein, which recruits a specific histone methyltransferase. In fission yeast, a putative DCAF protein, Dos1/Raf1/Clr8/Cmc1 15–18, interacts with Cul4, Rik1, and Clr4 (histone H3K9 methyltransferase) to regulate the heterochromatin formation. It was reported that Dos1, which is associated with Rik1 and important for the function of the Clr4-Rik1 complex, is essential for the recruitment of Clr4 in the RNAi-dependent heterochromatin pathway [17].

To find out how *N. crassa* DCAF proteins participate in histone H3K9 methylation and DNA methylation, we explored potential DCAF proteins that might interact with Cul4-DDB1 E3 ubiquitin ligase. We found that DCAF26 was associated with DDB1 and was essential for H3K9 trimethylation and DNA methylation. Protein interaction experiments revealed that, as with DIM-5 [19], DCAF26 preferentially interacted with neddylated Cul4 species. Interactions between DDB1 and DIM-5 were dependent on DCAF26; this indicates that DCAF26 served as a link between Cul4-DDB1 and DIM-5 in this pathway, thus regulating H3K9 trimethylation. Interestingly, in fission yeast, the loss of H3K9 methylation in *dos1* deletion mutants is similar to that in *rik1* and *clr4* mutants, and more severe than in RNAi mutants [17], suggesting that Dos1 is required for histone H3 modification in the same pathway as Rik1 and Clr4.

### Interaction between DDB1 and DCAF26 is essential for H3K9 trimethylation and DNA methylation

Immunoprecipitation experiments revealed that DCAF26 bridges the Cul4-DDB1 complex to DIM-5 and functions in the same DNA methylation pathway in *N. crassa*. If DCAF26 protein interacts with DDB1, Cul4, and DIM-5 to form a complex, we would expect the association of DCAF26-DDB1 to play an essential role in the function of the Cul4-DDB1-DCAF26-DIM-5 complex. Recent studies showed that many DCAF proteins contain two conserved WDXR motifs that are the key interacting modules with DDB1 in Cul4-DDB1 E3 ubiquitin ligases [3], [4], [13], [29], [30]. In this study, we showed that deletion of a region with a WDXR motif in DCAF26, but not the single-amino acid substitution of WNVR976A, eliminated DCAF26-DDB1 interactions and H3K9 trimethylation and DNA methylation in vivo, indicating that the interaction between DCAF26 and DDB1 is required for H3K9 trimethylation and DNA methylation. Although several studies showed that the arginines in conserved WDXR motifs in DCAF proteins are required for the association between DCAFs and DDB1, some DCAFs that lack the conserved motifs can still interact with DDB1 and Cul4 proteins [8], [9]. Interestingly, *S. pombe* Dos1/Raf1/Clr8/Cmc1 contains two conserved WDXR motifs [4]; however, mutagenesis studies of these motifs to examine the interactions with Rik1 protein have not yet been performed. Alignment of these two motifs and adjacent regions in *S. pombe* Dos1 with the corresponding region in *N. crassa* showed that they share a high similarity, suggesting that this region is important for interactions with DDB1. Indeed, the results of our deletion and mutagenesis studies revealed that the WDXR-containing WD3 region, but not the arginine residue, was necessary for productive assembly with DDB1 into a functional H3K9 methyltransferase complex under physiological conditions. In addition, we found that the WDXR-containing WD3 region was not required for interactions between DCAF26 and DIM-5, and confirmed that the association of DCAF26-DDB1 was essential for recruiting DIM-5 to regulate H3K9me3. Thus, the Cul4-DDB1-DCAF26 complex serves to recruit the histone methyltransferase DIM-5 for H3K9 trimethylation, which serves as a mark for DNA methylation.

### The substrate(s) of the Cul4-DDB1-DCAF26 ubiquitin ligase

Our results indicate that DCAF26 protein was necessary for recruiting DIM-5 into the Cul4-DDB1 complex. In addition, both DCAF26 and DIM-5 preferentially interacted with neddylated Cul4 species. These data suggest that the Cul4-DDB1-DCAF26 complex is required to recruit DIM-5 or to ubiquitinate a specific substrate(s) to perform its epigenetic functions. In fission yeast, in vitro data show that H2B can be polyubiquitinated by a purified complex containing Cul4-Rik1-Raf1-Raf2-Clr4 [16]. In mammalian cells, the putative targeting substrate of Cul4-DDB1-WDR5 is histone H3 [13]. In human cells, Cul4-DDB1^DDB2^ promotes the ubiquitination of histones H3 and H4 [31]. However, under normal growth conditions, the Cul4-DDB1-DCAF26 complex mainly recruits DIM-5 to regulate H3K9 trimethylation and DNA methylation. In contrast, histones H2B and H4 were copurified with ubiquitin, Cmc1, and Clr4 in Rik1 purification products and were suggested as the likely targets of Cul4-mediated ubiquitination in fission yeast [15]. In mammalian cells, XPC was identified as a substrate directly targeted by the DCAF protein DDB2 to the DDB1-DDB2-CUL4A-Rbx1 E3 complex [25]. In *S. pombe*, the DCAF protein Cdt2 is required for the degradation of Spd1 through ubiquitination by the Cul4-DDB1 E3 ligase [7]. However, no direct binding is observed between Cdt2 and its target protein Cdt1 in mammalian cells [29]. These results suggest that it may be difficult to identify the substrate of a specific DCAF by copurifying its interacting proteins. We tried to identify the substrate of the Cul4-DDB1-DCAF26 complex by purification of Myc-His-DCAF26 in *N. crassa*. In Myc-His-DCAF26 affinity purification products, Cul4, DDB1, DIM-5, and Nedd8 were copurified, strongly suggesting that they form an E3 ubiquitin ligase. Unlike the Rik1 purification in fission yeast, no ubiquitinated protein was detected in the purification. This result suggests that the substrate is ubiquitinated at low levels, such as mono-ubiquitination, by the Cul4-DDB1-DCAF26 complex, or that the ubiquitinated substrate is released immediately from its E3 complex. Alternatively, it is also possible that ubiquitinated protein(s) do not associate tightly with this complex during H3K9 methylation and DNA methylation. The activating signals of DNA methylation can trigger assembly of the Cul4-DDB1-DCAF26 complex, in which neddylated Cul4 promotes the complex formation with DDB1 and DCAF26, and then recruits DIM-5 at specific DNA regions for H3K9 trimethylation. This functional complex would link the initial signals of DNA methylation, and downstream epigenetic modifications on histones and DNA.

Together, our results suggest that in the DNA methylation process, the biochemical function of the Cul4-DDB1-DCAF26 complex is to recruit DIM-5 to control H3K9 methylation and DNA methylation in *N. crassa*. Because Cul4-DDB1 complexes are conserved in higher eukaryotes, we propose that analogous Cul4-DDB1-DCAF complexes may have a similar role in these organisms.

## Materials and Methods

### Strains and culture conditions

In this study, *87-3* (*bd a*) was used as the wild-type strain. All of the *dcaf* gene knockout mutants were newly generated from the *bd ku70^RIP^* genetic background strain and are listed in Table 1. The *ddb1^KO^*, *cul4^KO^*, *dim-5^KO^*, and *dim-2^KO^* strains generated previously [19] were also included in this study. The 301-6 (*bd*, *his-3*, *A*), *ddb1^KO^* (*bd*, *his-3*), and *dim-5^KO^* (*bd*, *his-3*) strains were used as the host strain for the *his-3* targeting constructs.

Liquid cultures were grown in minimal medium (1× Vogel's, 2% glucose). For QA-induced gene expression, 0.01 M QA (pH 5.8) was added into liquid medium containing 1× Vogel's, 0.1% glucose, and 0.17% arginine [32]. For the racetube assay, the medium contained 1× Vogel's, 0.1% glucose, 0.17% arginine, 50 ng/mL biotin, and 1.5% agar.

### Plasmid construction and creation of cotransformation strains

Full-length open reading frames (ORFs) and the 3′-UTR of all putative DCAF proteins were PCR amplified from genomic DNA and cloned into pqa-5Myc-6His. To study the interaction of Myc-tagged DCAF26 with Flag-tagged Cul4 or Flag-DIM-5, the Myc-tagged construct was introduced into transformants expressing Flag-Cul4 or Flag-DIM-5 by cotransformation with pBT6 (containing the benomyl resistance gene, obtained from the Fungal Genetics Stock Center). Western blot analyses using a monoclonal c-Myc antibody (9E10, Santa Cruz Biotechnology) were performed to identify the positive cotransformants. Immunoprecipitation assays using c-Myc antibody or Flag antibody (F3165-5MG, Sigma) were performed to test the interactions between Myc-tagged DCAF26 and Flag-Cul4 or Flag-DIM-5 in positive cotransformants. Protein extraction, quantification, western blot analysis, and immunoprecipitation assays were performed as described previously [32], [33].

### Generation of *dcaf* knockout mutants by gene replacement

Sixteen *dcaf* gene knockout mutants were generated following the knockout procedures described previously [19]. Briefly, the entire ORF knockout cassette of each gene was created by PCR. The *hph* gene replacement cassette was introduced into the *ku70^RIP^* strain by electroporation. The transformants with *hph* at the ORF of the targeted *dacf* gene locus were passaged on minimal slants with hygromycin for five generations. The homokaryotic knockout strains of these *dacf* genes were then obtained by conidia purification and confirmed by PCR analysis.

### DNA methylation analysis

The protocol of DNA methylation assay was the same as described previously [19]. Briefly, genomic DNA (200 ng) was digested with DpnII or BfuCI (Sau3AI) in 50 µL reaction system. No enzyme was added to the control samples. The PCR primers for *ku70*, ζ-η, Ψ63, and cen VII regions are listed in Table 2. PCR was performed using 1 µL of the digested DNA as a template in a 50 µL reaction system, with a program of 5 min at 94°C, followed by 31 cycles at 94°C (30 sec), 53°C (30 sec), and 72°C (1 min). The PCR products were resolved by electrophoresis on 2% agarose gels. Each experiment was performed independently at least three times.

**Table 2:** List of primers used in this study.

Target	Location	Range on Contig	Length	Primers	Sequence (5′ to 3′)	Purpose
*ku70*	Chromosome IV Contig 53	147012–147629	618 bp	*ku70.1F*	GAAGAATGGAAGAGAAGCACGG	DNA methylation H3K9me3 ChIP
				*ku70.2R*	TGGGAGATAATTCGCTGCTGC	
		147686–148623	938 bp	*ku70.3F*	ATGATTCGGGCAATGGCACAG	DNA methylation H3K9me3 ChIP
				*ku70.4R*	GACATATTGCTCCTCGCTAGG	
		148653–149358	706 bp	*ku70.5F*	GCAGAAGCTCCTCAAAGATGAC	DNA methylation H3K9me3 ChIP
				*ku70.6R*	AAGTCTCTCAACTAGCTCAGCC	
ζ-η	Chromosome I Contig 9	945683–945860	178 bp	*ζ-η.1F*	ACACTTAGGATTCGCTAATCGTC	DNA methylation H3K9me3 ChIP
				*ζ-η.2R*	GTACGATCCTATCGGCTTAC	
Ψ63	Chromosome IV Contig 49	26128–26691	564 bp	*Ψ63.1F*	ACATACGACCATACCCACTGG	DNA methylation H3K9me3 ChIP
				*Ψ63.2R*	GGTCTAGGGATATATTGGGAGG	
centromere	Chromosome VII Contig 55	144426–145009	584 bp	*cenVII.FF*	CTACCTATCTGGCTCTAGCTTTC	DNA methylation H3K9me3 ChIP
				*cenVII.FR*	CTCGAAATCGGGCCCTCTAATC	
*hH4*	Chromosome II Contig 5	428931–429351	421 bp	*hH4.5′*	AACCACCGAAACCGTAGAGGGTAC	H3K9me3 ChIP
				*hH4.3′*	ATCGCCGACACCGTGTGTTGTAAC	

### ChIP analysis

For the ChIP assay, tissues were fixed in minimal media containing 1% formaldehyde for 10 min at 25°C. ChIP was performed using 8 µL of antibody to H3K9me3 (07-442, Upstate Biotechnology) or 10µL of antibody to H3 (06-755, Upstate Biotechnology) for 6 mg/ml protein. After washing with 70% ethanol, extracted DNA pellets were resuspended in 30 µL of double-distilled water, and 0.5 µL of the DNA solution was used for PCR. The primers for *ku70*, ζ-η, Ψ63, and hH4 regions are listed in Table 2. PCR conditions were as follows: 5 min at 94°C, 26–30 cycles at 94°C (30 sec), 53°C (30 sec), and 72°C (1 min). Different PCR cycles were tested to ensure that DNA amplification was within the exponential amplification range. PCR products were resolved by electrophoresis on 2% agarose gels. ChIP assays with anti-Myc antibody or no antibody were used as negative controls. Each experiment was independently performed at least three times.

### Western blot analysis of histone H3 and trimethylated H3K9

*N. crassa* histone proteins were extracted from the wild-type strain, *dcaf26^KO^*, *ddb1^KO^*, *cul4^KO^*, and *dim-5^KO^* strains, as described previously [34]. Equal amounts of histone protein extracts were loaded onto 15% SDS-PAGE gels. Western blot analysis was performed using antibodies against trimethylated H3 Lys9 (07-442 Upstate Biotechnology) or H3 (06-755 Upstate Biotechnology).

### Purification of Myc-His-DCAF26 from *N. crassa*

*dcaf26^KO^* Myc-His-DCAF26 strain and the wild-type strain (as the negative control) were cultured for approximately 20 hr in constant light (LL) in liquid medium containing QA (0.01 M QA, 1× Vogel's, 0.1% glucose, and 0.17% arginine). Approximately 10 g of tissue from each strain grown in LL was harvested. The purification procedure was the same as described previously [22]. Fractions containing purified Myc-His-DCAF26 were immunoprecipitated by adding 20 µL of c-Myc monoclonal antibody-coupled agarose beads (9E10AC, Santa Cruz Biotechnology). The precipitates were analyzed by SDS-PAGE (2%–20%), which was subsequently silver stained following the manufacturer's instructions (ProteoSilver Plus, Sigma). The specific bands were excised and subjected to tryptic digestion and liquid chromatography–mass spectrometry/mass spectrometry analysis (LC-MS/MS).

## Supporting Information

Figure S1 Specificity of the DDB1 antibody. Western blot analysis of DDB1 protein in the wild-type and *ddb1^KO^* strains using anti-DDB1 serum (A) or DDB1 antibody after depletion using tissue of the *ddb1^KO^* strain (B) as the primary antibody. Asterisks indicate nonspecific bands detected by our DDB1 antibody.
(0.11 MB TIF)

Figure S2 Interactions between the other 11 DCAFs and DDB1. c-Myc antibody was used for immunoprecipitation, followed by western blot analysis using the DDB1 and c-Myc antibodies. The wild-type strain was used as a negative control, and DCAF11 was used as the strong interaction control (lane 1).
(0.52 MB TIF)

Figure S3 DNA methylation analysis in other *dcaf^KO^* strains. DNA methylation in the wild-type strain (WT) and *ku70^RIP^*, *dim-2^KO^*, and *dcaf^KO^* strains on (A) the ζ-η region and (B) the ψ63 region detected by methylation-sensitive restriction digestion. The knockout mutants were in the *bd ku70^RIP^* background. Genomic DNA digested by 5mC-sensitive BfuCI (*B*) or its 5mC-insensitive isoschizomer, DpnII (*D*), was amplified by PCR with the labeled primers. Untreated genomic DNA as template for PCR was used as the control (*CD* or *CB*).
(0.86 MB TIF)

Figure S4 DIM-7 required for H3K9 trimethylation and DNA methylation in *N. crassa*. (A) Dense, cauliflower-like growth pattern of *dim-7^KO^* strain (*bd ku70^RIP^* background) on plate with minimal media (30°C, 32 hr). (B) DNA methylation in the wild-type strain (WT) and demethylation in the *dim-7^KO^* strain at the ψ63 region. (C) Loss of histone H3K9 trimethylation at ψ63 region in the *dim-7^KO^* strain. Levels of H3K9 trimethylation at the ψ63 region were determined by ChIP assay. Myc antibody was used as a negative control. H3 antibody was used as the positive control and as the control for integrity of the nucleosome structure. (*D*) Western blot analysis of global H3 and H3K9 trimethylation in the wild-type and *dim-7^KO^* strains.
(0.67 MB TIF)
