# Supplementary figures and images for: DCAF26, an Adaptor Protein of Cul4-Based E3, Is Essential for DNA Methylation in Neurospora crassa
Source: PLoS Genet. 2010 Sep 23;6(9):e1001132. doi: 10.1371/journal.pgen.1001132 (PMC2944796; doi:10.1371/journal.pgen.1001132)

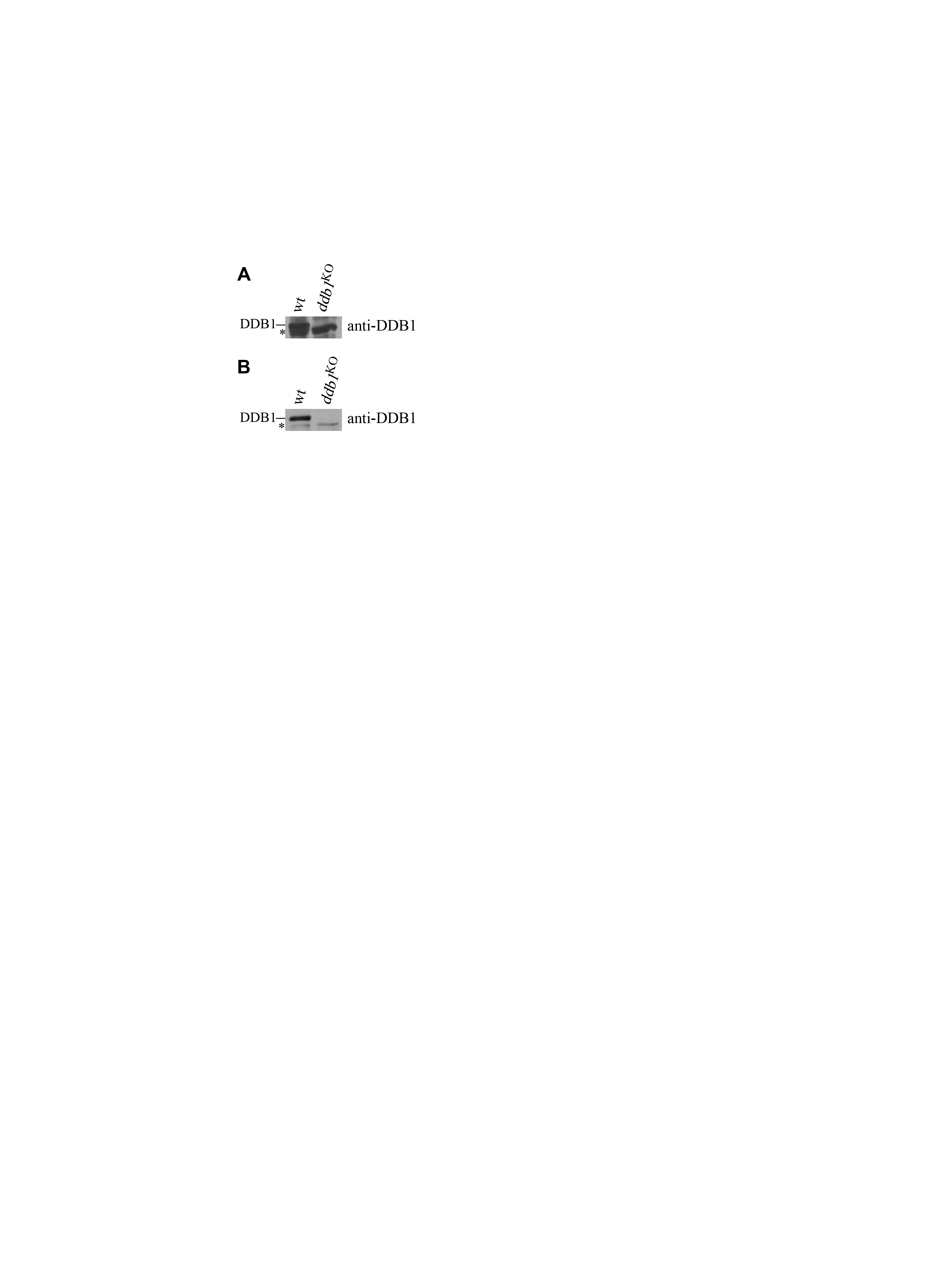

Supplement: Figure S1 — Specificity of the DDB1 antibody. Western blot analysis of DDB1 protein in the wild-type and ddb1KO strains using anti-DDB1 serum (A) or DDB1 antibody after depletion using tissue of the ddb1KO strain (B) as the primary antibody. Asterisks indicate nonspecific bands detected by our DDB1 antibody. (0.11 MB TIF) [file pgen.1001132.s001.tif]

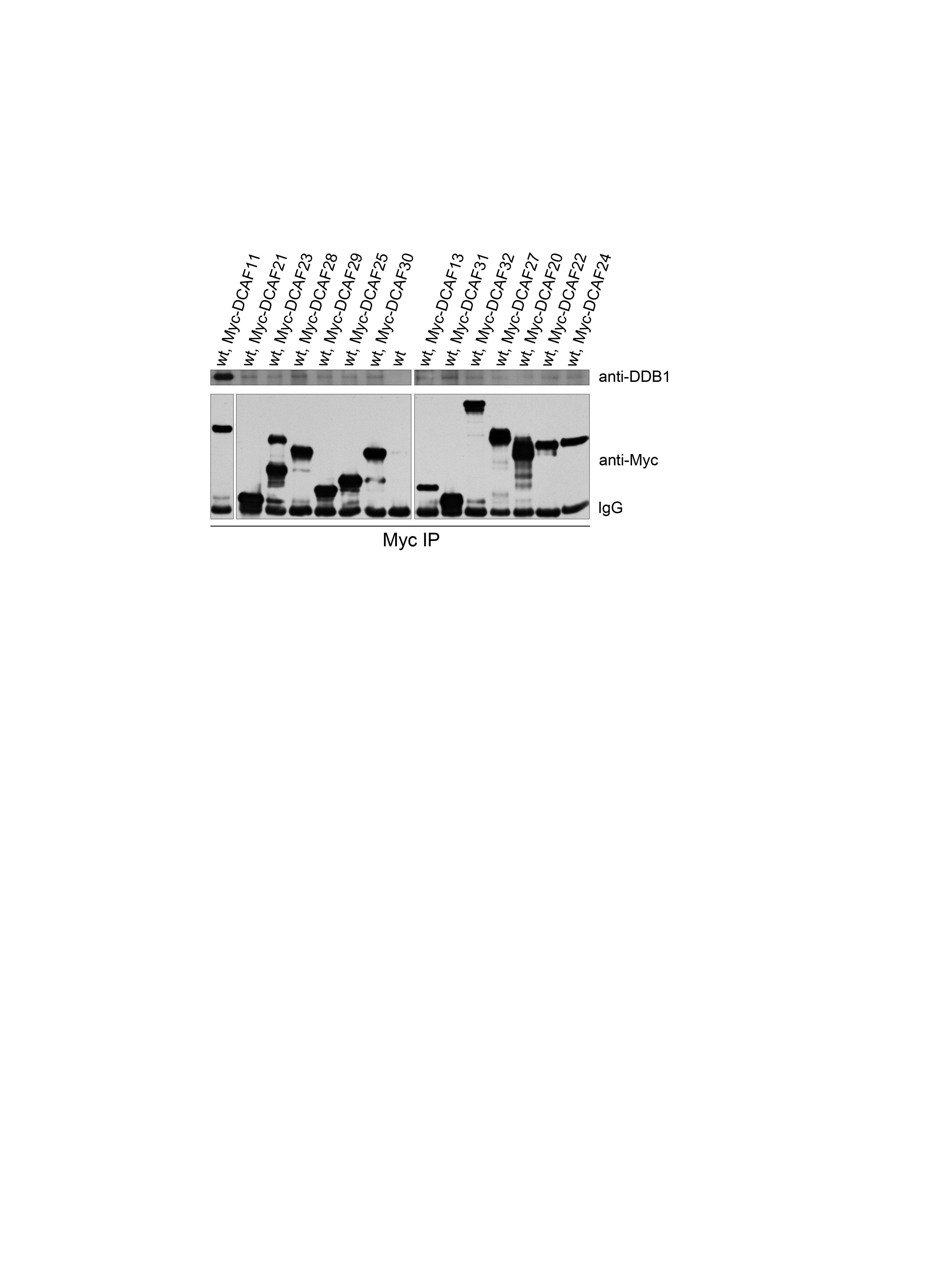

Supplement: Figure S2 — Interactions between the other 11 DCAFs and DDB1. c-Myc antibody was used for immunoprecipitation, followed by western blot analysis using the DDB1 and c-Myc antibodies. The wild-type strain was used as a negative control, and DCAF11 was used as the strong interaction control (lane 1). (0.52 MB TIF) [file pgen.1001132.s002.tif]

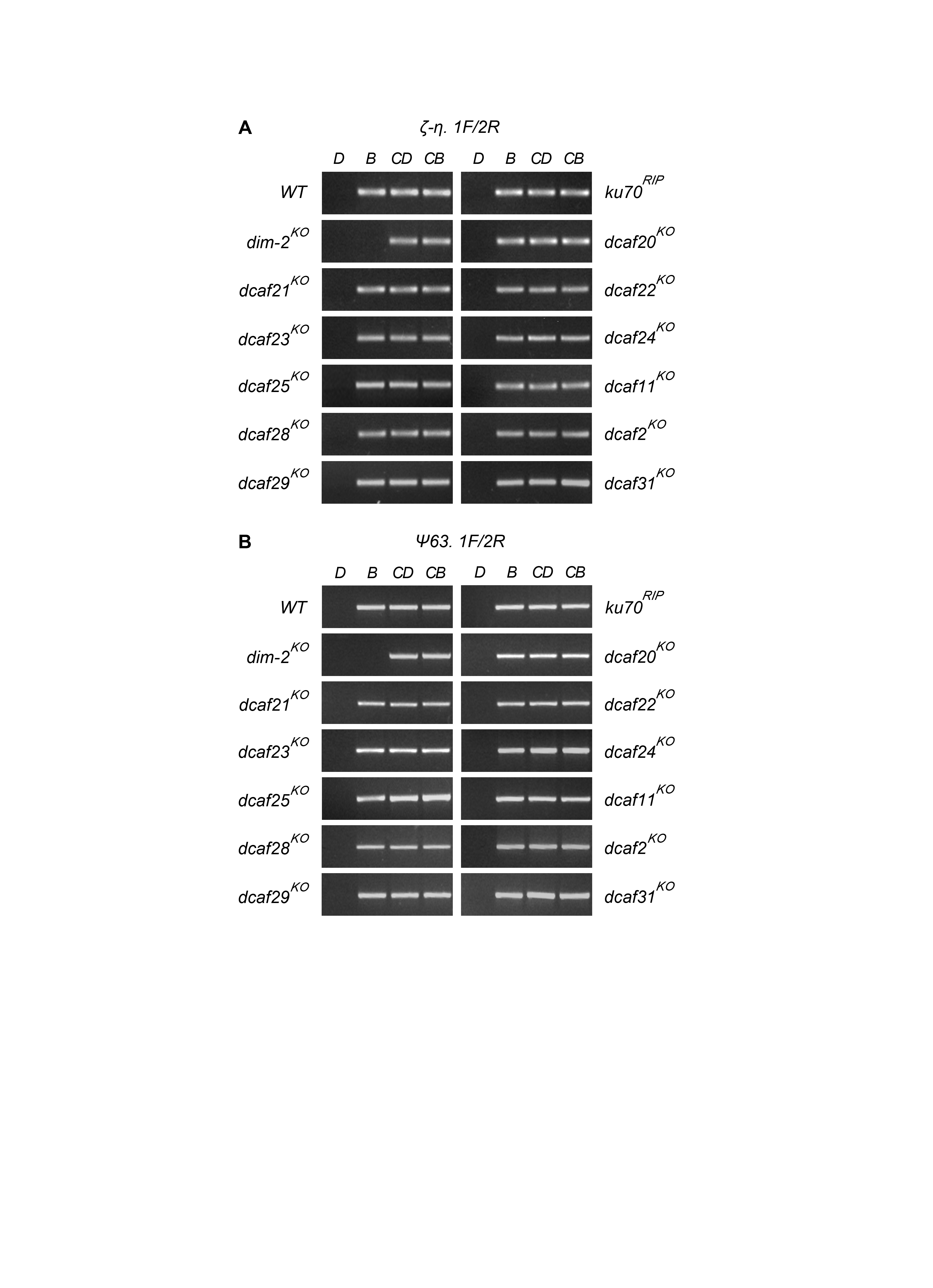

Supplement: Figure S3 — DNA methylation analysis in other dcafKO strains. DNA methylation in the wild-type strain (WT) and ku70RIP, dim-2KO, and dcafKO strains on (A) the ζ-η region and (B) the ψ63 region detected by methylation-sensitive restriction digestion. The knockout mutants were in the bd ku70RIP background. Genomic DNA digested by 5mC-sensitive BfuCI (B) or its 5mC-insensitive isoschizomer, DpnII (D), was amplified by PCR with the labeled primers. Untreated genomic DNA as template for PCR was used as the control (CD or CB). (0.86 MB TIF) [file pgen.1001132.s003.tif]

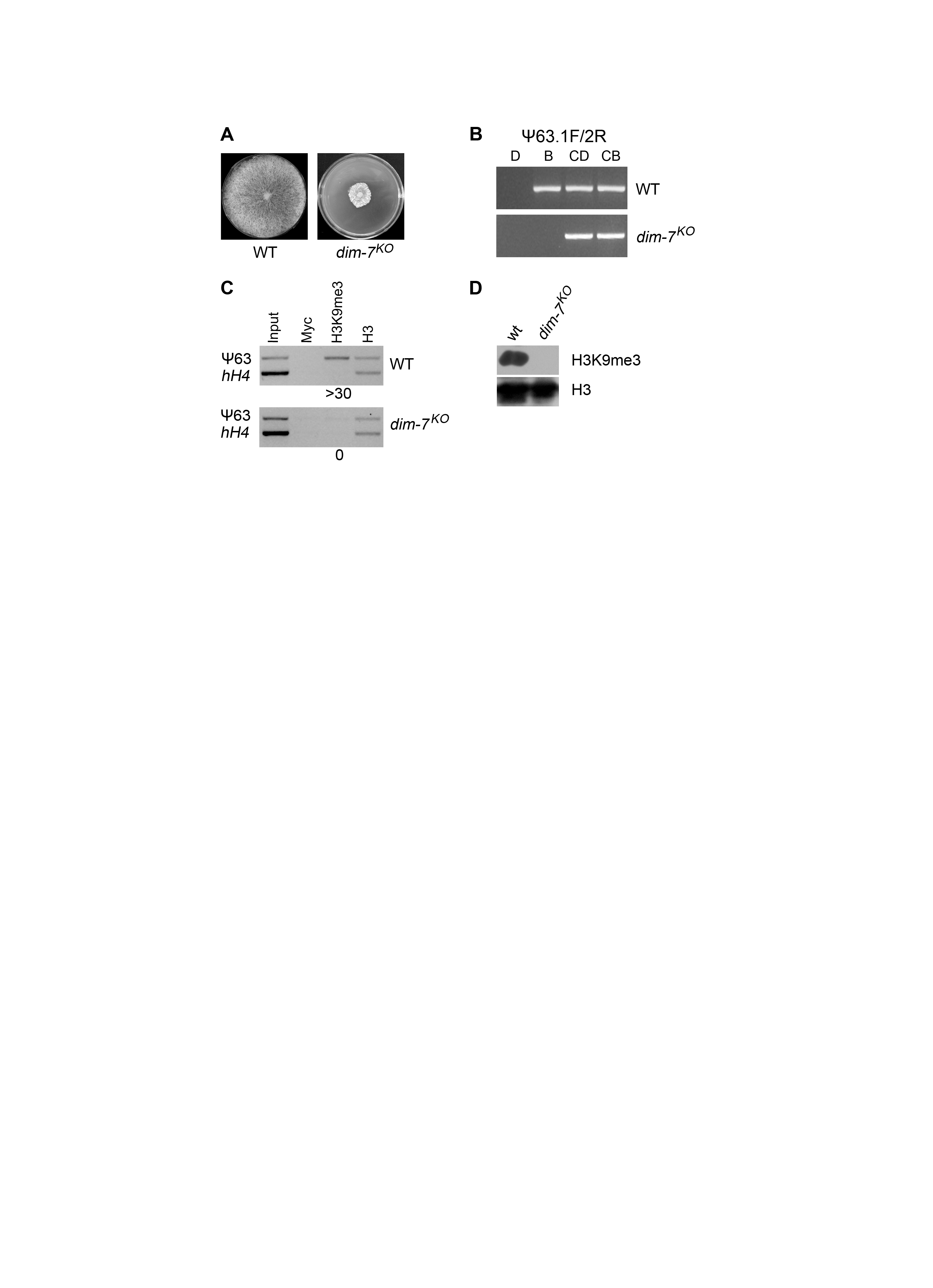

Supplement: Figure S4 — DIM-7 required for H3K9 trimethylation and DNA methylation in N. crassa. (A) Dense, cauliflower-like growth pattern of dim-7KO strain (bd ku70RIP background) on plate with minimal media (30°C, 32 hr). (B) DNA methylation in the wild-type strain (WT) and demethylation in the dim-7KO strain at the ψ63 region. (C) Loss of histone H3K9 trimethylation at ψ63 region in the dim-7KO strain. Levels of H3K9 trimethylation at the ψ63 region were determined by ChIP assay. Myc antibody was used as a negative control. H3 antibody was used as the positive control and as the control for integrity of the nucleosome structure. (D) Western blot analysis of global H3 and H3K9 trimethylation in the wild-type and dim-7KO strains. (0.67 MB TIF) [file pgen.1001132.s004.tif]
